# Cutaneous Squamous Cell Carcinoma Arising Within a Previously Irradiated Mycosis Fungoides Lesion

**DOI:** 10.7759/cureus.73071

**Published:** 2024-11-05

**Authors:** Shannon C South, Kimberly A Kluglein, Bryce Demoret, Richard Miller

**Affiliations:** 1 Osteopathic Medicine, Lake Erie College of Osteopathic Medicine, Bradenton, USA; 2 Osteopathic Medicine, Dr. Kiran C. Patel College of Osteopathic Medicine, Nova Southeastern University, Clearwater, USA; 3 Dermatology, Hospital Corporation of America/University of South Florida Morsani College of Medicine: Largo Medical Center Program, Largo, USA

**Keywords:** cutaneous squamous cell carcinoma, cutaneous t-cell lymphoma, mycosis fungiodes, non-melanoma skin cancer, radiotherapy

## Abstract

Mycosis fungoides (MF), a form of cutaneous T-cell lymphoma (CTCL), increases the risk of other malignancies. A common and effective treatment for patients with MF is radiotherapy (RT), which itself also increases the risk of malignancies. One such malignancy that may result from both MF and RT is cutaneous squamous cell carcinoma (cSCC). cSCC is the second most common skin cancer in the United States. Prognosis may range from excellent to grim and correlates with the presence of established high-risk features. This case follows a 64-year-old male with a prior diagnosis of MF treated with localized radiation approximately 17 years ago who was found to have a large biopsy-proven moderately differentiated cSCC arising in the scar of an MF radiation site. This patient required general surgery to excise a 10 cm x 20 cm x 2 cm area of skin to have clear deep and peripheral margins. This case underscores the importance of regular skin checks in patients with multiple risk factors for developing cSCC, especially in those with a history of MF, RT, or both to early identify complications before they cause disfigurement or advanced disease.

## Introduction

Mycosis fungoides (MF) is the most common form of cutaneous T-cell lymphoma (CTCL), accounting for approximately 60% of cases [1]. MF arises from the clonal proliferation of neoplastic T-lymphocytes that selectively infiltrate and persist within the skin [2]. Patients with early-stage disease may present with eczema-like lesions or widespread erythema, whereas those with advanced disease may develop fungating tumors with lymph node, blood, or organ involvement [3]. As a highly radiosensitive condition, MF responds exceptionally well to radiotherapy (RT), making it a frequently used treatment modality across all stages of the disease due to its strong therapeutic efficacy [4]. 

MF increases the risk of other malignancies, including non-melanoma skin cancers (NMSC) such as cutaneous squamous cell carcinoma (cSCC) [5]. cSCC is the second most common type of skin cancer in the United States, with over one million cases diagnosed each year [6]. The most well-documented risk factor for cSCC is cumulative ultraviolet sun exposure on unprotected skin [7]. Additional risk factors for the development of cSCC are Fitzpatrick skin types I-III, age, with an age of onset in the mid-60s, male sex, immunosuppression and organ transplant recipients, human papillomavirus, chronic scarring conditions, familial cancer syndromes, and environmental exposures such as arsenic [8-10]. Solid organ transplant recipients (SOTR) have been found to have an elevated risk of cSCC compared to the general population, with an increased risk of 65-250 times that of non-SOTR, with thoracic organ transplants carrying the highest risk [11]. Certain cancers have also been associated with cSCC, such as chronic lymphocytic leukemia (CLL) carrying an eight-fold increased risk [12]. Some prognostic factors to developing advanced or metastatic disease include positive borders, primary vs recurrent, growth rate, site of prior RT, lymphatic, or vascular involvement, with cSCC arising from ulcers, burn scars and radiation dermatitis carrying a 26% risk of metastasis [13]. Rarely, SCC has developed in lesions of MF, potentially in relation to treatment of MF with ultraviolet radiation, topical chemotherapies such as nitrogen mustard, and systemic immunosuppressive agents [14-16]. 

In this discussion, we present the case of a 64-year-old man with a history of MF treated with localized radiation, who developed moderately differentiated squamous cell carcinoma (SCC) in the irradiated area more than a decade later.

## Case presentation

**Figure 1:**
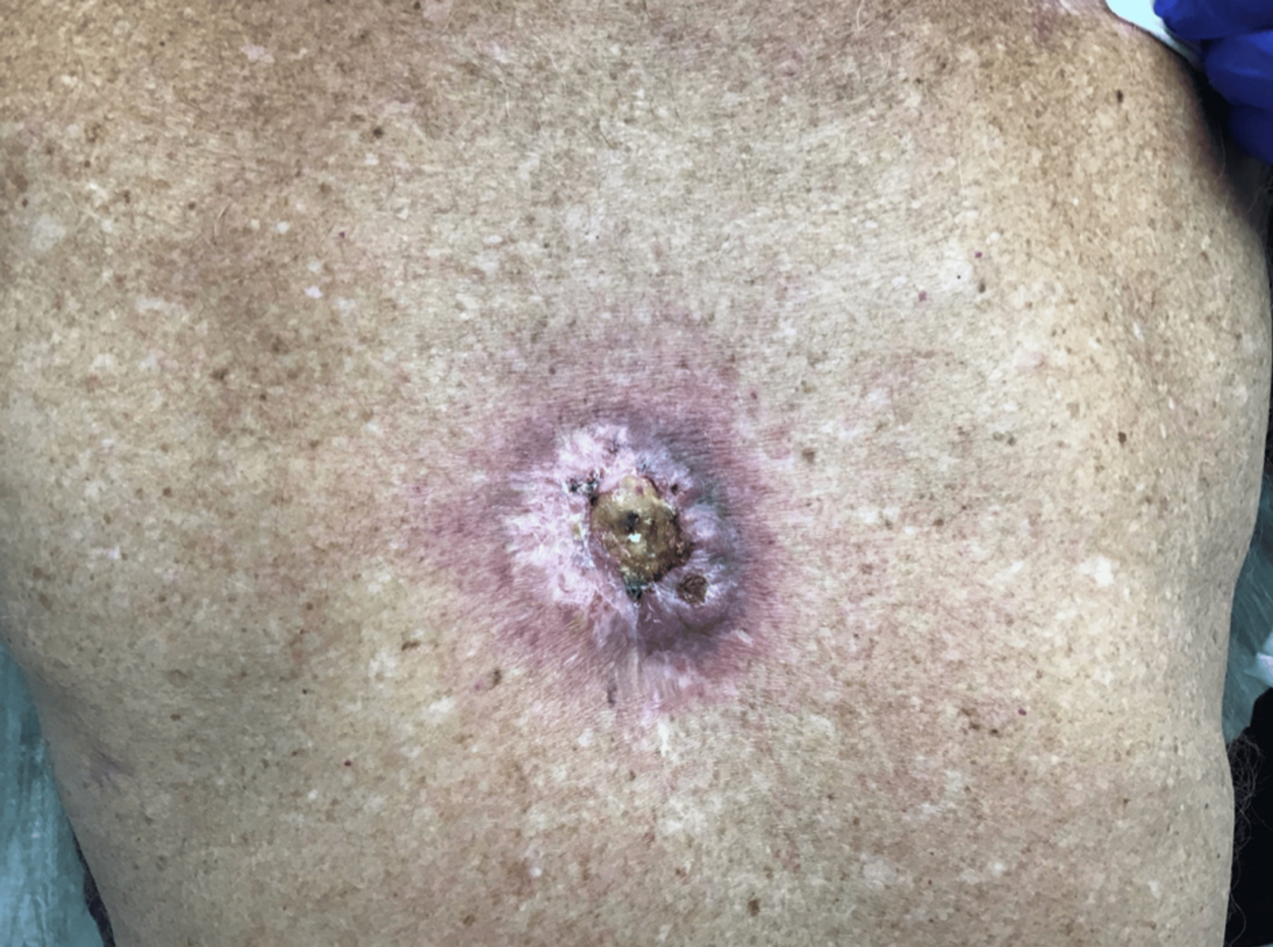
An erythematous, violaceous nodular plaque with central ulceration and purulent drainage overlying a previously irradiated MF lesion MF: Mycosis fungoides

A 64-year-old white male (Fitzpatrick skin type II) with a past medical history of MF presented to the outpatient dermatology clinic for evaluation of a lesion on his mid upper back which had been present for several months. The lesion appeared as an erythematous, violaceous nodular plaque with central ulceration and purulent drainage (Figure 1).

The lesion was observed within a scar which was secondary to localized radiation in 2006 and 2007 for treatment of the patient’s MF. The patient denied associated or systemic symptoms and there was no appreciable lymphadenopathy on the physical exam. Of note, this patient has had a biopsy proven SCC in situ treated with electrodessication and curettage (ED&C) in this same location several years prior.

A shave biopsy was performed to distinguish between recurrent CTCL, NMSC, and chronic radiation-induced skin ulcer. Histopathological analysis revealed an atypical, moderately differentiated squamous cell proliferation invading the dermis accompanied by a dermal inflammatory cell infiltrate. These findings were consistent with a diagnosis of invasive moderately differentiated SCC. Due to the large size, the patient was referred to general surgery for excision and staging. The lesion measured 8 cm x 8 cm, and the excised skin and subcutaneous tissue extended approximately 10 cm x 20 cm x 2 cm. The defect was closed using an elliptical incision with layered, linear closure. Both deep and peripheral margins were free of residual disease. Utilizing the Brigham and Women’s Hospital (BWH) staging system, our patient fortunately only met criteria for one of the four high-risk features, size > 2 cm, indicating stage T2a disease. 

## Discussion

MF is a CTCL frequently treated with RT [4]. While effective, both low and moderate doses of ionizing radiation have been associated with an increased risk of NMSCs including SCC. The highest incidence of SCC is observed within the radiation field as well as sun-exposed areas. The risk of developing SCC can persist for over 40 years following radiation treatment, with the likelihood of occurrence peaking around 20 years post exposure [17].

Some studies have proposed additional mechanisms for SCC development in MF patients. For instance, Muradova et al. documented a case in which superficially invasive SCC developed within an active MF patch in a patient who had not received any prior local or systemic therapy [14]. Similarly, Le et al. described a patient with untreated plaque/patch-stage MF who subsequently developed multiple moderately differentiated SCCs [16]. These observations suggest that SCC may arise in MF patients even in the absence of RT or systemic treatment, indicating a potential role in immune-mediated mechanisms, such as changes in the early tumor microenvironment. Notably, the risk of developing cSCC increases with more advanced forms of MF, which presents a key limitation in comparing these studies as the patients were at varying stages of the disease [16]. 

Consistent with the current literature on documented risk factors, our patient developed SCC approximately 17 years after completing localized radiation treatment, specifically within the irradiated area. Notably, despite having previously undergone ED&C for SCC in situ at the same location, the patient progressed to invasive SCC. It is unlikely that a single factor led to the development of SCC in this patient; rather, a combination of multiple risk factors, including a history of radiation, CTCL, sun exposure, and chronic scarring is likely to have contributed.

In their 1989 review, Edwards et al. examined 66 patients who developed SCC in skin previously damaged by burns or radiation [18]. They discovered that SCCs in these cases are locally aggressive and exhibit distinct characteristics compared to SCCs that result from cumulative sun exposure. Survival in these patients depended largely on the complete surgical removal of all detectable disease [18]. Consequently, excision of SCC developing in previously burned or irradiated skin should be managed with increased vigilance, prompting our decision to treat with aggressive wide surgical margins greater than 2 cm to achieve total histologic clearance. 

A recent study evaluating SCC BWH T2a tumors specifically found that tumors who met one major and at least one minor criterion were found to have an increased risk for poor outcomes. However, fortunately for our patient, he only met one major criteria (size > 40 mm) and no other major or minor criteria. Pertinent conditions not met in our case included invasion below the dermis into the subcutaneous fat, large or small caliber perineural involvement, lymphovascular involvement, or moderate to poor differentiation on histology [19]. 

Collectively, these findings underscore the critical importance of regular dermatologic screenings for all patients with a history of MF. Heightened awareness is particularly important for individuals who were treated with RT. Since the risk of developing SCC peaks years following radiation, long-term monitoring is essential to detect early signs of skin cancer. Furthermore, it is crucial to educate patients about their heightened risk for NMSCs, encourage regular self-examinations, and promote photoprotective measures including daily use of broad-spectrum sunscreen. 

Further research is needed to determine the precise incidence of cSCC arising within MF lesions. Additionally, it is crucial to explore and identify effective strategies for reducing malignancy risk in these patients. By deepening our understanding of these relationships, we may enhance patient management and improve outcomes.

## Conclusions

MF is a common form of CTCL. Its treatment typically involves topical phototherapy, systemic immunosuppression, and, most commonly, ultraviolet radiation. While radiation is therapeutic, it is a known risk factor for cSCC, with the risk often peaking years after treatment. Furthermore, immune-mediated factors may increase risk of secondary malignancies in patients with MF, although the precise mechanisms behind this increased susceptibility remains elusive. Consequently, regular and comprehensive skin examinations in addition to routine age-appropriate cancer screenings are crucial for these patients given their elevated risk.
